# Relationships between parameters of EEG and glutamate-ergic system activity in patients with depressive-delusional disorders

**DOI:** 10.1192/j.eurpsy.2025.1690

**Published:** 2025-08-26

**Authors:** A. F. Iznak, ?. V. Iznak, A. F. Beresneva, O. K. Savushkina, I. S. Boksha

**Affiliations:** 1 Laboratory of Neurophysiology; 2 Laboratory of Neurochemistry, Mental Health Research Centre, Moscow, Russian Federation

## Abstract

**Introduction:**

Activity of the glutamate neurotransmitter system contribute to the development of many mental disorders, in particular of depression and schizophrenia. While the glutamate is the principal excitatory neurotransmitter in the brain there are poor data on its relationships with EEG.

**Objectives:**

The aim of the study was to search for possible relationships between parameters of EEG and glutamate dehydrogenase (GDH) activity in patients with depressive-delusional disorders.

**Methods:**

The study involved 28 female in-patients aged 16-35 years (mean age 22.0 ± 8.1 years) with depressive-delusional disorders in the frames of schizophrenia (F20.01, by ICD-10). Pre-treatment multichannel resting EEG recordings with spectral power analysis in narrow frequency sub-bands were performed in all patients. Patients were divided into two groups with relatively “normal” (n=18) and “slow” (n=10) EEGs. “Slow EEG” group statistically differed (p<0.05) from “normal EEG” group by greater spectral power values in theta2 (6-8 Hz) EEG sub-band in frontal-central-temporal regions of the left hemisphere. GDH enzymatic activity was measured in platelets’ extracts from blood samples by spectrophotometric kinetic method and was assessed by the rate of NAD•H absorption decrease at 340 nm. The descriptive statistics and the rank correlation analysis (Spearman) were used for statistical processing of EEG and neurochemical data.

**Results:**

“Slow EEG” and “normal EEG” groups did not statistically differ (p>0.05) in age, HDRS and PANSS scores, while the clinical severity was somewhat greater in “slow EEG” group. Nevertheless, “slow EEG” and “normal EEG” groups apparently differed in correlation structure between EEG and GDH activity parameters. Thus, in “slow EEG” group values of GDH activity correlated positively (p<0.05÷0.01) with values of spectral power in delta (2-4 Hz), theta1 (4-6 Hz) and theta2 (6-8 Hz) EEG sub-bands. In “normal EEG” group values of GDH activity did not correlate with any studied EEG parameters.

**Conclusions:**

Greater GDH activity in “slow EEG” group of patients with depressive-delusional disorders have to provoke the glutamate mediated excitation deficit reflected in EEG slowing. The predominance of this phenomenon in the left hemisphere may underlie some features of the clinical conditions in these patients and need further studying.

**Disclosure of Interest:**

None Declared

